# Supplementary material for: Development of loop-mediated isothermal amplification (LAMP) assay for rapid and direct screening of yellowfin tuna (Thunnus albacares) in commercial fish products
Source: PLoS One. 2022 Oct 12;17(10):e0275452. doi: 10.1371/journal.pone.0275452 (PMC9555631; doi:10.1371/journal.pone.0275452)
Supplement: S1 Data — (PDF) [file pone.0275452.s001.pdf]

Table 1s Manufacturers of fish samples tested with *cytB* LAMP and real-time PCR

Fish species	Sample No.	Manufacturer
Gadus chalcogrammus	ANT10	Gut & Günstig, Edeka Zentrale Stiftung & Co. KG, Hamburg, Germany
Gadus chalcogrammus	ANT11	REWE GmbH, Köln, Germany
Gadus chalcogrammus	ANT12	Netto Marken-Discount Stiftung & Co. KG, Maxhütte-Haidhof, Germany
Gadus morhua	ANT5	Edeka Zentrale Stiftung & Co. KG, Hamburg, Germany
Gadus morhua	ANT6	Edeka Zentrale Stiftung & Co. KG, Hamburg, Germany
Gadus morhua	ANT7	OceanSea, Lidl Stiftung & Co. KG, Neckarsulm, Germany
Gadus morhua	ANT8	Followfish, followfood GmbH, Friedrichshafen, Germany
Gadus morhua	ANT9	Netto Marken-Discount Stiftung & Co. KG, Maxhütte-Haidhof, Germany
Katsowonus pelamis	T15	BioGourmet GmbH, Erdmannshausen, Germany
Katsowonus pelamis	T16	Saupiquet Deutschland GmbH, Krefeld, Germany
Katsowonus pelamis	T17	Edeka Zentrale Stiftung & Co. KG, Hamburg, Germany
Katsowonus pelamis	T18	Followfish, followfood GmbH, Friedrichshafen, Germany
Katsowonus pelamis	T20	Saupiquet Deutschland GmbH, Krefeld, Germany
Katsowonus pelamis	T23	Saupiquet Deutschland GmbH, Krefeld, Germany
Onchorhynchus keta	ANT1	FEMEG Produktions- und Vertriebs GmbH, Rehna, Germany
Onchorhynchus keta	ANT2	Gut & Günstig, Edeka Zentrale Stiftung & Co. KG, Hamburg, Germany
Onchorhynchus nerka	ANT4	Followfish, followfood GmbH, Friedrichshafen, Germany
Salmo salar	ANT3	Gut & Günstig, Edeka Zentrale Stiftung & Co. KG, Hamburg, Germany

Thunnus albacares	T3	Deutsche See GmbH, Bremerhaven, Germany
Thunnus albacares	T4	Guba Trade GmbH, Schenefeld, Germany
Thunnus albacares	T5	I. Schroeder KG (GmbH & Co.), Hamburg, Germany
Thunnus albacares	T6	Deutsche See GmbH, Bremerhaven, Germany
Thunnus albacares	T7	I. Schroeder KG (GmbH & Co.), Hamburg, Germany
Thunnus albacares	T8	Guba Trade GmbH, Schenefeld, Germany
Thunnus albacares	T25	Followfish, followfood GmbH, Friedrichshafen, Germany
Thunnus albacares	T26	Followfish, followfood GmbH, Friedrichshafen, Germany
Thunnus albacares	T27	Followfish, followfood GmbH, Friedrichshafen, Germany
Thunnus albacares	T28	Followfish, followfood GmbH, Friedrichshafen, Germany
Thunnus albacares	T29	Followfish, followfood GmbH, Friedrichshafen, Germany
Thunnus albacares	T30	Followfish, followfood GmbH, Friedrichshafen, Germany
Thunnus albacares	T31	Edeka Zentrale Stiftung & Co. KG, Hamburg, Germany
Thunnus albacares	T32	Edeka Zentrale Stiftung & Co. KG, Hamburg, Germany
Thunnus albacares	T34	Edeka Zentrale Stiftung & Co. KG, Hamburg, Germany
Thunnus albacares	T35	REWE GmbH, Köln, Germany
Thunnus albacares	T37	REWE GmbH, Köln, Germany
Thunnus albacares	T38	REWE GmbH, Köln, Germany

Figure 1s: Sequences alignment of the cytochrome *b* gene of *Thunnus albacares* (JN086153.1), *Thunnus albacares* (EF392629.1), *Thunnus albacares* (EF141179.1), *Oncorhynchus keta* (MN011567.1), *Katsuwonus pelamis* (EF141175.1), *Gadus morhua* (EU492141.1), *Gadus chalcogrammus* (AB078151.1) and *Salmo salar* (BT044011.1). Identical nucleotide sequences are indicated by dots. The position of the LAMP oligonucleotide primers are marked.

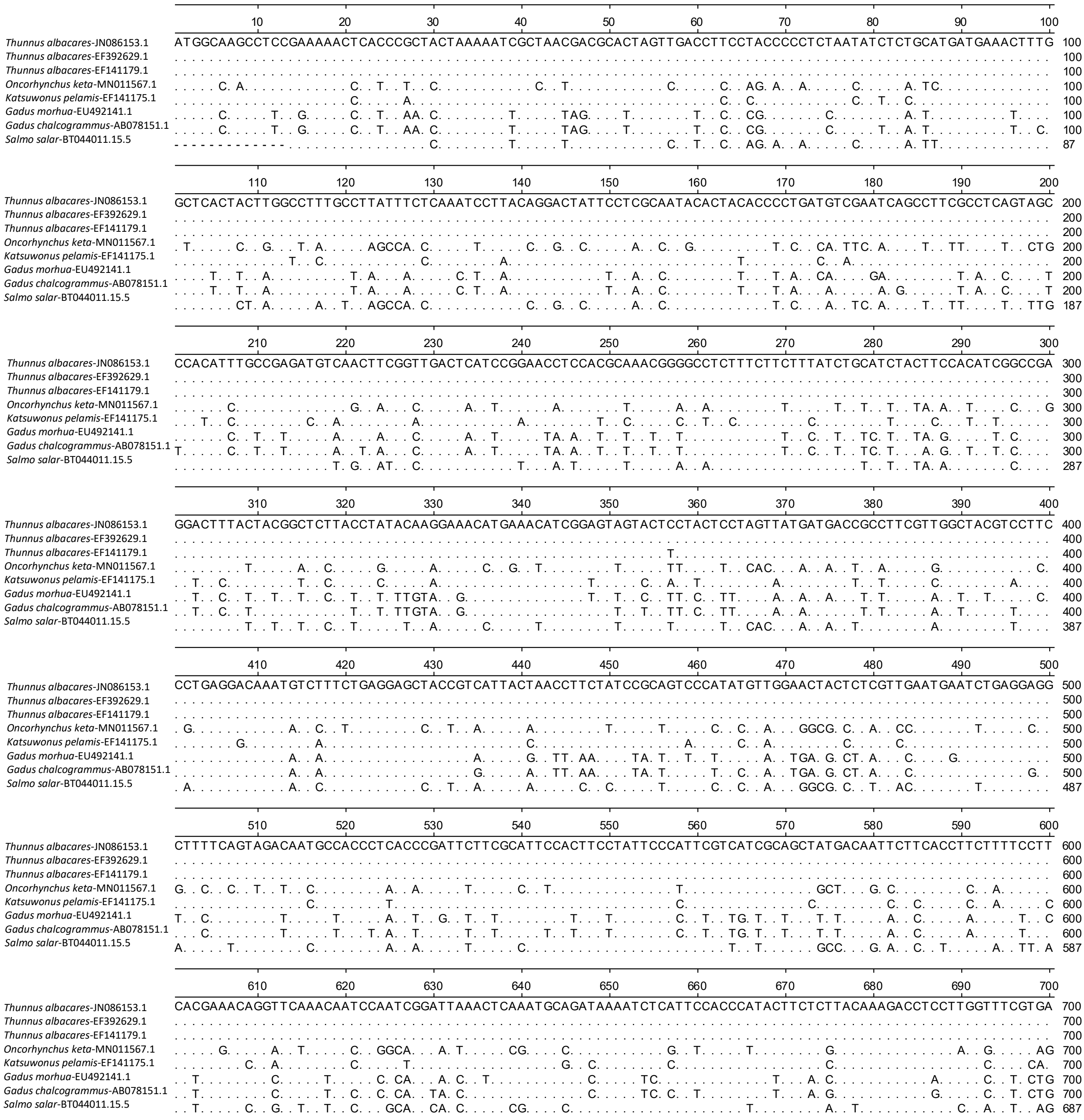

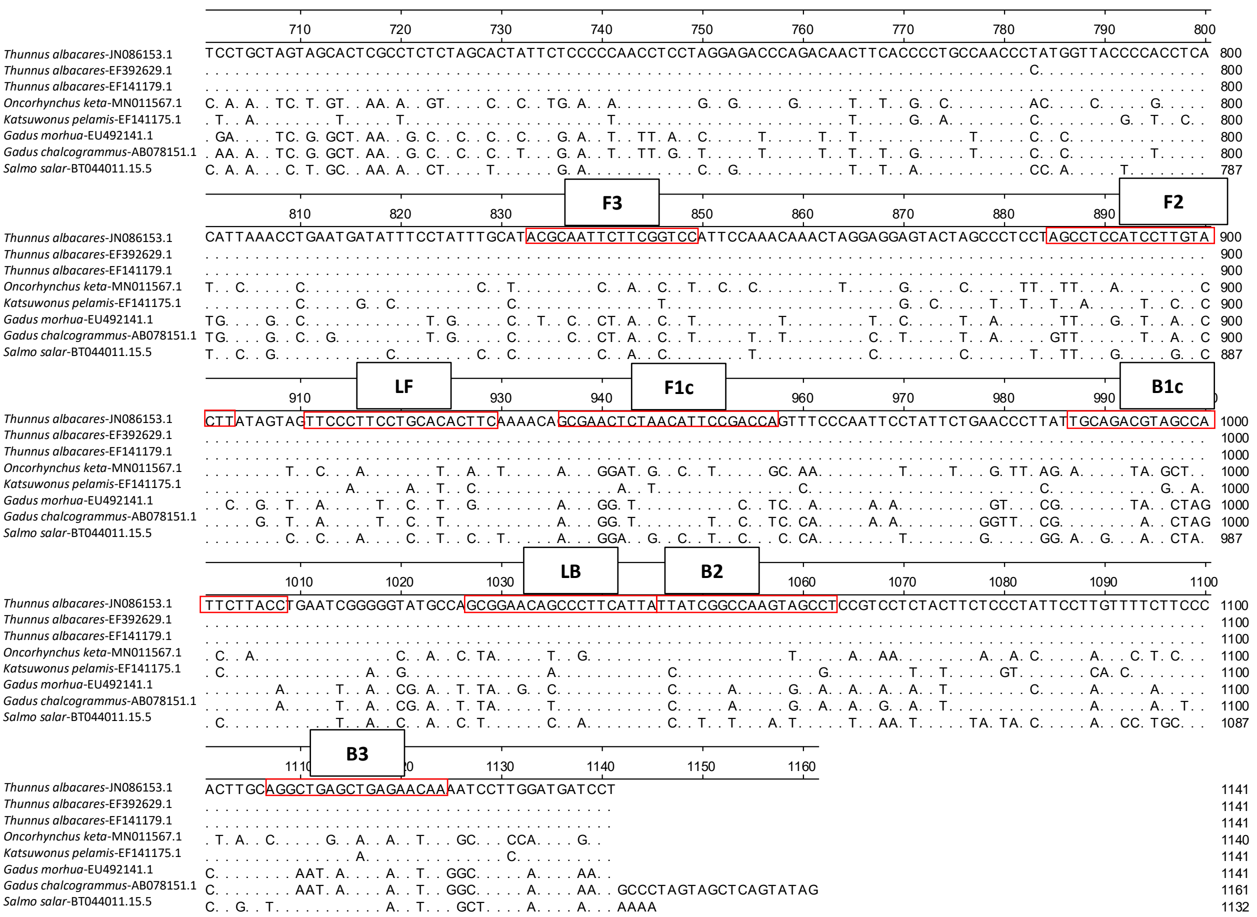

F3
F2
LF
F1c
B1c
LB
B2
B3
Thunnus albacares-JN086153.1
Thunnus albacares-EF392629.1
Thunnus albacares-EF141179.1
Oncorhynchus keta-MN011567.1
Katsuwonus pelamis-EF141175.1
Gadus morhua-EU492141.1
Gadus chalcogrammus-AB078151.1
Salmo salar-BT044011.15.5
TCCTGCTAGTAGCACTCGCCTCTCTAGCACTATTCTCCCCAACCTCCTAGGAGACCCAGACAACTTCACCCCTGCCAACCCTATGGTTACCCCACCTCA 800
CATTAAACCTGAATGATATTTCCTATTTGCATACGCAATTCTTCGGTCCATTCCAAACAAACTAGGAGGAGTACTAGCCCTCCTAGCCTCCATCCTTGTA 900
CTTATAGTAGTTCCCTTCCTGCACACTTCAAAACAGCGAACTCTAACATTCCGACCAGTTTCCCAATTCCTATTCTGAACCCTTATTGCAGACGTAGCCA 1000
TTCTTACCTGAATCGGGGGTATGCCAGCGGAACAGCCCTTCATTATTATCGGCCAAGTAGCCTCCGTCCTCTACTTCTCCCTATTCCTTGTTTTCTTCCC 1100
ACTTGCAGGCTGAGCTGAGAACAAAATCCTTGGATGATCCT 1141
