# Supplementary figures and images for: Development of loop-mediated isothermal amplification (LAMP) assay for rapid and direct screening of yellowfin tuna (Thunnus albacares) in commercial fish products
Source: PLoS One. 2022 Oct 12;17(10):e0275452. doi: 10.1371/journal.pone.0275452 (PMC9555631; doi:10.1371/journal.pone.0275452)

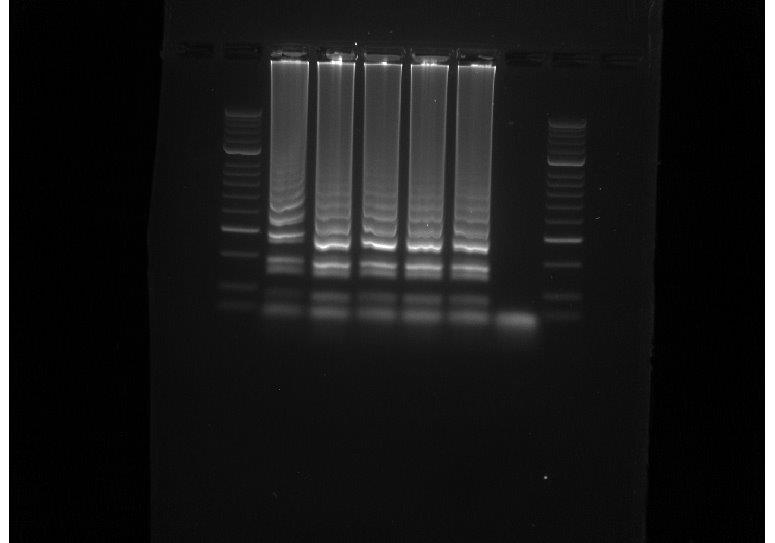

Supplement: S1 Raw images — (TIF) [file pone.0275452.s002.tif]
