# Supplementary material for: Development of loop-mediated isothermal amplification (LAMP) assay for rapid and direct screening of yellowfin tuna (Thunnus albacares) in commercial fish products
Source: PLoS One. 2022 Oct 12;17(10):e0275452. doi: 10.1371/journal.pone.0275452 (PMC9555631; doi:10.1371/journal.pone.0275452)
Supplement: Attachment — Submitted filename: Review comments.docx [file pone.0275452.s003.docx]

1.This work needs a deep review of English throughout the document.

2.In this study, the authors describe an assay for yellowfin tuna with a lower limit of detection of 540 fg/μl and good specificity as well as providing a rapid DNA extraction method.

3.In my opinion, the authors needed to benchmark a recognized assay for 36 samples, and fluorescent quantitative PCR（qPCR） was a good choice.

4. LAMP as a common method for isothermal amplification needs to be compared with other isothermal amplification methods such as RPA, RCA, CPA, SDA, and HDA, and the authors need to add this section in the Introduction and Discussion section.

5.The authors mentioned that the detection target sequences were compared, and the results of this section need to be presented as supplementary material.

6.Changes need to be made to Figure 4, and I think the left Marker is best deleted.
